# The climate crisis and planetary health in teaching

**DOI:** 10.3205/zma001621

**Published:** 2023-05-15

**Authors:** Christoph Nikendei, Susanne J. Kühl, Till Johannes Bugaj

**Affiliations:** 1 Heidelberg University Hospital, Department of General Internal Medicine and Psychosomatics, Heidelberg, Germany; 2 University Ulm, Institute of Biochemistry and Molecular Biologie (iBMB), Ulm, Germany

## Editorial

Future human mental and physical health depends on addressing the climate crisis.

So simple.

So complex.

And yet the climate crisis threatens to fade into the background in light of the numerous other crises that are taking place around the world, each of which confronts us with barely manageable tasks [1]: The Corona crisis, Ukraine crisis, economic crisis, migration crisis.

We – as public health representatives and workers – have different roles and responsibilities in the face of the central threat to humanity posed by the climate crisis:

- As practitioners, we will be faced with an increase in mental and physical illnesses that are directly and indirectly related to global warming. Many insightful textbooks now exist and are at our disposal for orientation [1], [2], [3].
- As bearers of responsibility and role models within our society, we have the scientific and communicative competencies to inform people about the climate crisis and its effects, to initiate transformation processes [1], [4], and to join interest groups [5], [6], [7].
- As representatives of the health care system, we are, on the one hand, guarantors of human health, and, on the other hand, we represent a system that is responsible for 4.4% of global CO_2_ emissions. In order to have a realistic chance of overcoming the climate crisis, we must also drastically reduce CO_2_ emissions in the healthcare system. Here, we can take action in the form of sustainability initiatives [8] or in the context of programs that monitor and attempt to reduce emitted CO_2_ [9].
- Last but not least, we as lecturers and teachers are challenged to promote and deepen knowledge and action skills around the climate crisis and planetary health [10].

This special issue “Climate crisis and planetary health in teaching” aims to raise awareness and motivate the development and establishment of courses and curricula on the climate crisis and planetary health. At the same time, many medical faculties already have thematically overloaded curricula, and the implementation of the new medical licensing regulations is just around the corner. Therefore, university and teaching hospitals are facing major challenges. Nevertheless, integrating the issues surrounding the climate crisis and planetary health, as well as professionalizing related education, training, and continuing education, is an imperative.

This special issue provides insights into current developments, some of which are “in their infancy” or already highly differentiated – a valuable thematic mosaic:

Schwienhorst-Stich et al. [11] and Bökels et al. [12] deal with an inventory of courses on planetary health in the national and international context. A needs assessment at the student level is presented by Rybol et al. [13]. Initiatives of (online) electives on environment, climate, and planetary health and climate communication in climate science are presented by Flägel et al. [14], Fülbert et al. [15], Schmid et al. [16], Müller et al. [17] and Lemke et al. [18]. Grundacker et al. [19] initiated a course on medical ecology, Jonas et al. [20] a seminar on future scenarios. Innnovative ideas for raising awareness related to climate change and planetary health are presented by Praschinger et al. [21] and Straßer et al. [22]. Impulses for planetary health in continuing education and training are presented by Rieser et al. [23].

## Competing interests

The authors declare that they have no competing interests. 
